# Homology judgements of pre-evolutionary naturalists explained by general human shape matching abilities

**DOI:** 10.1038/s41598-023-39036-2

**Published:** 2023-07-28

**Authors:** Ulrich E. Stegmann, Filipp Schmidt

**Affiliations:** 1 School of Divinity, History, Philosophy and History of Art, University of Aberdeen, Aberdeen, UK; 2 Experimental Psychology, Justus Liebig University Giessen, Otto-Behaghel-Str. 10F, 35394 Giessen, Germany; 3 Center for Mind, Brain and Behavior (CMBB), Philipps-University Marburg and Justus Liebig University, Giessen, Germany

**Keywords:** Psychology, Human behaviour

## Abstract

Many biological homologies were discovered before Darwin and without agreed criteria. Paradigmatic examples include the phylogenetic homology of mammalian forelimb bones and the serial homology of floral organs in waterlilies. It is generally assumed that perceiving similarities intuitively was the first step towards establishing morphological homologies. However, this assumption has never been tested. We hypothesize that pre-evolutionary naturalists relied on the well-established ability of humans to find visual correspondences between differently shaped objects. By using images of homologous organs and applying an experimental paradigm from cognitive psychology, we found that (1) na﻿ïve participants utilised this ability when identifying “corresponding” locations. In addition, (2) these locations were statistically indistinguishable from the locations that pre-evolutionary naturalists and contemporary experts considered homologous. Furthermore, (3) presenting na﻿ïve participants with images of intermediate organs influenced their correspondence judgements. This influence was in line with historical reports according to which intermediate organs facilitated the pre-evolutionary recognition of homologies.

## Introduction

**Figure 1:**
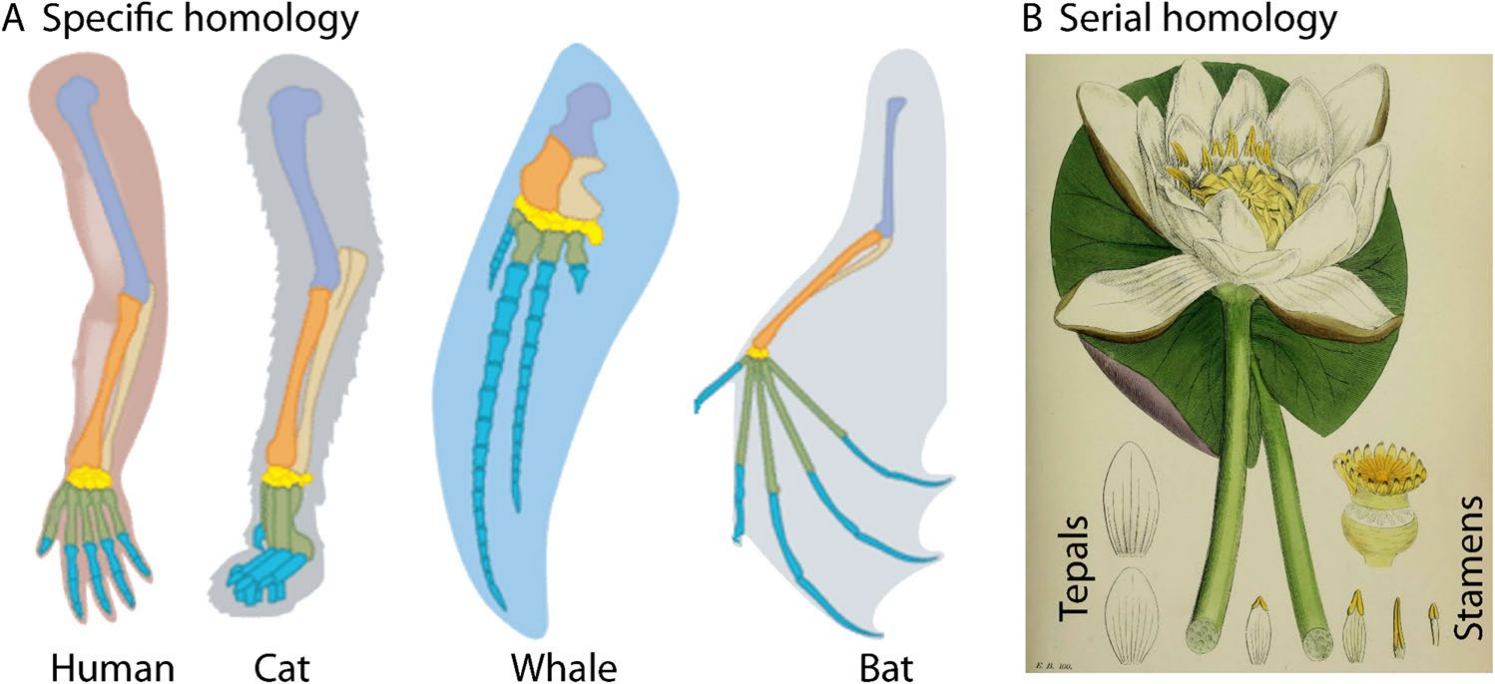
Two kinds of pre-evolutionary homology. (**A**) The forelimbs of different mammals with colors indicating the different bones (humerus: purple, ulna: beige, radius: orange, and wrist bones: yellow). Adapted from image by Christopher AuYeung/CK-12 Foundation, published under Creative Commons License CC BY-NC 3.0. (**B**) Floral organs of the White Waterlily (*Nymphaea alba* L.): white tepals (bottom left), stamens (bottom right), and transitional forms (either side of the right leaf stem). Image from^11^.

Comparing natural objects to one another is one of the oldest practices of the natural sciences. In biology, it led to the recognition of homologies, or basic identity, of biological structures. Although homology definitions and criteria are still contested (e.g.^1^), it is widely accepted that homologies are biological characters that evolved from a common ancestor (e.g.^2^) and/or share certain developmental properties^3^. Yet many homologies were discovered decades before Darwin and before their ontogenetic development had been investigated. For example, the homology of mammalian forelimbs (Fig. 1A) was recognized as early as 1680, when the flipper of a small whale species was found to contain the same bones as a human arm^4^. In botany, eighteenth century naturalists promoted the current view that the floral organs of flowering plants are transformed leaves^5–7^ (Fig. 1B). At the time, naturalists mostly compared gross morphological structures of adult organisms^8^ and evolution was controversial. Moreover, the concepts of homology and analogy had not yet been distinguished clearly, and homology criteria not yet established. Both were subsequent developments during the first decades of the nineteenth century^8–10^.

How did pre-evolutionary naturalists make discoveries that, decades later, were found to be compatible with evolutionary theory and are still widely accepted today? One hypothesis is that they followed a familiar pattern of observation, comparison, and subsequent reasoning. The botanist De Candolle^12^, for instance, believed that biological sameness is a deliberate inference from observable facts. However, it is commonly assumed that homologizing began with something more like an intuition, a pre-theoretical impression of similarity^13,14^. The perception of similarity leads to a conjecture of homology (“primary homology”;^15^). Although this hypothesis has never been tested, advances in cognitive psychology render it plausible.

Humans have a highly developed ability to find visual correspondences between differently shaped objects. This ability has been demonstrated in experimental psychology using dot matching paradigms. Observers are presented with two differently shaped objects side by side, one of which has a dot. They are then asked to identify the dot’s “corresponding location” on the second object by placing another dot on the latter (e.g.^16–22^). The objects presented to observers can be two distinct objects or the same object under, say, different viewpoints. Such studies have shown that observers quickly select what they perceive to be corresponding locations and, moreover, that they agree on the correspondences of even minute shape details (high inter-individual consistency). This suggests the operation of specific and spontaneous perceptual organization processes^17^.

Pre-evolutionary naturalists may have relied on the same abilities: highly developed yet spontaneous shape-matching, in addition to or instead of deliberate comparison based on explicit criteria. This hypothesis generates several predictions.

If pre-evolutionary naturalists used spontaneous shape-matching to find identical organs and their parts, then biologically untrained, contemporary observers who use their own shape-matching abilities should agree among themselves about which biological structures they perceive as (visually) corresponding. And they should do so quickly, without much deliberation. Furthermore, the structures that contemporary observers perceive as corresponding should in fact be identical to the structures that pre-evolutionary naturalists judged as belonging to the same kind. If they were different, then pre-evolutionary naturalists may have shared our shape-matching abilities, but there would be no evidence that these abilities led to discovering homologies.

Another prediction concerns intermediate forms. When pre-evolutionary naturalists found that two particular structures are the same, they often studied additional structures with intermediate properties^8,12^. Having access to intermediate forms should therefore also make a difference to the correspondence judgements of contemporary observers. For instance, observers might judge different structures as corresponding, depending on whether they perceive intermediates. If intermediates do affect which structures are perceived as corresponding, then we predict that the intermediate-based structures are the ones identical to pre-evolutionary correspondences. This is because pre-evolutionary correspondences were also based on intermediates.

In sum, the hypothesis that pre-evolutionary naturalists matched structures wholly or partly by spontaneous shape-matching generates the following predictions:

### P1

Participants make quick and mutually consistent correspondence judgements.

### P2

Participants’ correspondence judgments differ depending on whether they are shown intermediate forms.

### P3

The locations that participants identify as (visually) corresponding match the locations pre-evolutionary naturalists believed to be identical.

We test these predictions with floral organs and arm bones, two paradigmatic examples from the history of homology research. Pre-evolutionary naturalists identified what are now recognised as homologies within both groups (Supplementary Discussion, 1.1 and 1.2). Furthermore, the examples illustrate the two traditional types of homology, as first defined by Owen^23^. The bones were from four different mammals (dolphin, whale, otter, monkey), thus illustrating homologies between structures in different species (“specific homology”^23^, p. 7) (Fig. 1A). The floral organs were from the White Waterlily, exemplifying homologies between organs in a single individual (“serial homology”^23^, p. 8) (Fig. 1B). The correspondence judgments of untrained participants were determined by using the dot matching paradigm. Each participant was presented with dots on one organ (the base stimulus) and asked to identify the “corresponding” locations on another (the test stimulus). The dot matching paradigm allows us to tie our results to previous work in cognitive psychology.

## Materials and methods

### Participants

Participants (n = 20, 17 female, 3 male, mean age = 26.4, standard deviation = 5.5) were recruited through a university mailing list. All participants gave informed consent before the experiments and all methods were performed in accordance with the Declaration of Helsinki. Procedures were approved by the Local Ethics Committee of the Department of Psychology and Sports Sciences of the Justus Liebig University of Giessen (LEK-2017-0046).

Participants lacked university-level training in biology and were not given any information about the stimulus objects and how they relate, including any reference to homology. Furthermore, no information was provided about the “correspondence” they were asked to indicate. To further mitigate the role of circumstantial biological knowledge, we tested participants on small parts of organs, familiarity with which would have required advanced, specialist training in morphology.

### Stimuli and experts

As stimuli we used images of floral organs and mammalian arm bones (see below for details). Pre-evolutionary correspondences among floral organs and arm bones, respectively, were identified by studying the publications of pre-evolutionary naturalists and asking experts to locate the actually homologous parts. We first read the primary and secondary literature to determine, in as much detail as possible, which parts of these organs were identical according to pre-evolutionay naturalists (Supplementary Discussion, 1.1 and 1.2). We then asked experts, not to reconstruct pre-evolutionary homologies, but to determine the homologous locations according to the current state of knowledge and their own scientific assessment. Experts were shown the same base stimuli, intermediates forms, and test stimuli as used in the experiments with untrained participants. Experts placed dots in appropriate locations on the test images to record their judgements. This approach allowed detecting potential disparities among contemporary experts as well as between experts and pre-evolutionary naturalists. Importantly, the procedure allows representing pre-evolutionary correspondences, about which experts and pre-evolutionary naturalists agree, at the level of individual dots. This, in turn, enables us to quantitatively compare the correspondence judgements of contemporary observers, which are based on shape-matching, with those of pre-evolutionary naturalists.

#### Waterlily (serial homology)

**Figure 2:**
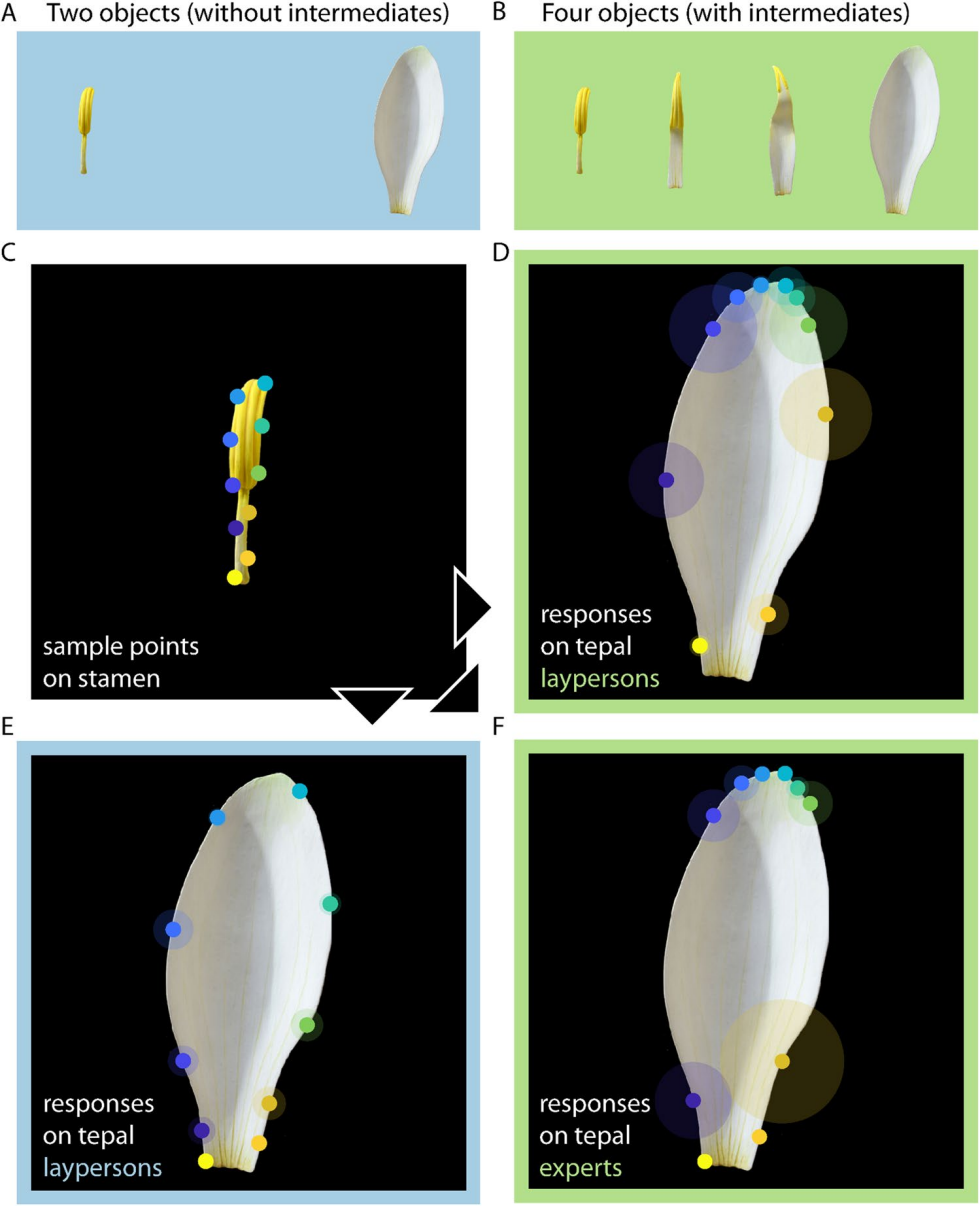
Waterlily stimuli and corresponding dot matching results. We tested two conditions, (**A**) with two objects (i.e. without intermediates; blue background) and (**B**) with four objects (i.e. with intermediates; green background). In both, participants were presented with (**C**) ten probe locations on the stamen. The tepal is shown with corresponding median responses of (**D**) naïve participants (four objects), (**E**) naïve participants (two objects), and (**F**) experts (group 2, four objects). The circles show the average distance between responses for each location, as a measure of consistency between individuals. Stimuli were photographs of plastic models housed in the Botanic Garden and Botanical Museum Berlin (“Übergangsformen von Staubblättern zu Blütenhüllblättern”, published under Creative Commons License CC-BY-SA 3.0).

As an example for serial homology, we used images of the stamen, tepal, and intermediate forms (petaloid stamens) of the White Waterlily (*Nymphaea alba* L.) (Fig. 2B). We obtained expert judgements from André Chanderbali (Department of Biology, University of Florida), Bruce Kirchoff (Department of Biology, University of North Carolina at Greensboro), Rolf Rutishauser (Institute of Systematic & Evolutionary Botany, University of Zurich), Pam Soltis and Doug Soltis (both Florida Museum of Natural History and Department of Biology at the University of Florida), and Mi-Jeong Yoo (Department of Biology, Clarkson University).

#### Mammalian bones (specific homology)

**Figure 3:**
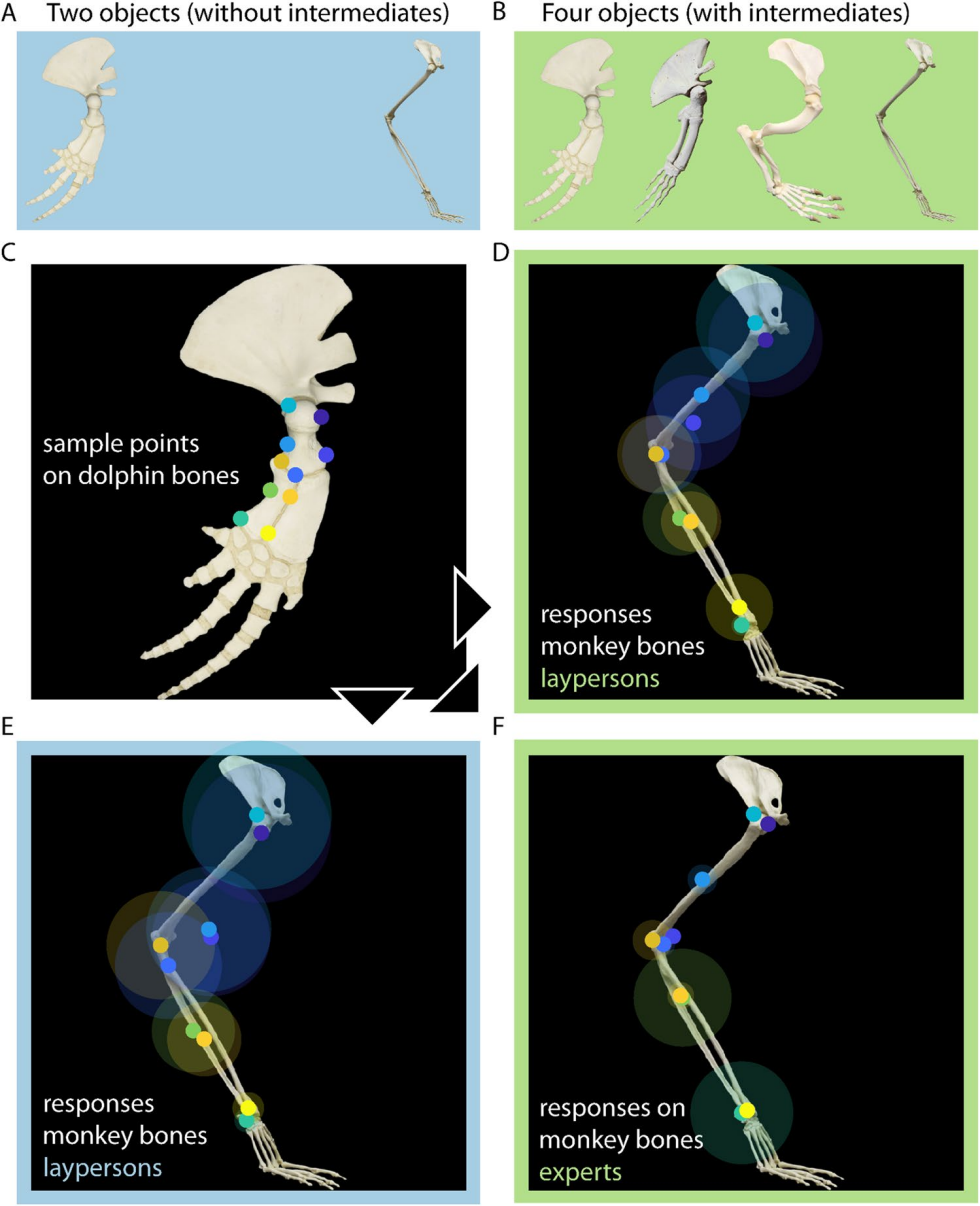
Mammalian bone stimuli and corresponding dot matching results. We tested two conditions, (**A**) with two objects (i.e. without intermediates; blue background) and (**B**) with four objects (i.e. with intermediates; green background; see text for species names). In both, participants were presented with (**C**) ten probe locations on the dolphin bones. The monkey bones are shown with corresponding median responses of (**D**) naïve participants (four objects), (**E**) naïve participants (two objects), and (**F**) experts (four objects). The circles show the average distance between responses for each location, as a measure of consistency between individuals. High-resolution photographs of dolphin and monkey bones were kindly provided by Jeff Shaw, Bone Clones, Inc., Chatsworth, California (https://boneclones.com). Photograph of fin whale bones by Frank Vincentz (published under Creative Commons License CC-BY-SA 3.0). Photograph of otter bones by Joe/Flickr (reprinted with permission).

As an example for specific homology, we used images of mammalian flipper and leg bones. Specifically, stimuli were photographs of the pectoral fin bones of a bottlenose dolphin (*Tursiops truncatus* Montagu) and a fin whale (*Balaenoptera physalus* L.) as well as the front leg bones of a North American river otter (*Lontra canadensis* Schreber) and a spider monkey (*Ateles paniscus* L.) (Fig. 3B). We obtained expert judgements from Lisa N. Cooper (Department of Anatomy and Neurobiology, Northeast Ohio Medical University), Spencer Hellert (Field Museum, Chicago), P. David Polly (Department of Earth and Atmospheric Sciences, Indiana University), and Heidi Schutz (Department of Biology, Pacific Lutheran University).

### Experimental procedure

Stimuli were cropped high-resolution photographs presented on a black background on a Dell U2412M monitor at a resolution of 1920 × 1200 pixels, controlled by Matlab using the Psychophysics Toolbox extension^24^. They were between 2.3°–11.4° (waterlily) or 13.7°–18.2° (mammalian bones) in width and 14.8°–27.0° (waterlily) or 31.1° (all mammalian bones) in height.

Each participant completed four blocks of the correspondence task, followed by three multiple choice questions, with two blocks each of the waterlily stimuli and the bone stimuli. Of those two blocks, one presented two stimuli (condition without intermediates), the other presented four stimuli (condition with intermediates). The order of blocks was counterbalanced across 10 participants. The other 10 participants only completed blocks without intermediates to test for effects of mere repetition.

In the block showing waterlily stimuli without intermediates, participants were presented with the stamen on the left and the tepal on the right side of the screen (Fig. 2A); in the corresponding block with intermediates, the petaloid stamens were presented between those two (Fig. 2B). Petaloid stamens combine tepal and stamen features to varying degrees. Pre-evolutionary naturalists therefore regarded them as intermediate forms (Supplementary Discussion, 1.1). In the block showing bone stimuli without intermediates, participants were presented with the flipper bones of a bottlenose dolphin on the left and the front leg bones of a spider monkey on the right side of the screen (Fig. 3A); in the corresponding block with intermediates, the flipper bones of a fin whale and the front leg bones of the North American river otter were presented between those two (Fig. 3B).

In each trial, a probe location was indicated by a red dot (bullseye with diameter = 0.9° and central dot = 0.1°) on the stimulus on the left side of the screen (waterlily stamen or dolphin bones), and the participants were asked to place a green dot (bullseye of the same size) “at the corresponding location” on the stimulus on the right side of the screen (waterlily tepal or river otter bones). After each response, the probe location was replaced by the next location, in random order. After participants responded to 10 probe locations (Figs. 2C and 3C), the next trial started. Each block consisted of three repetitions of the same trial, so that participants completed 12 trials (3 repetitions × 4 blocks).

The following multiple choice questions were asked after each block, referring to all three (identical) trials: Q1: What is the relationship between the left and the right object (multiple responses possible)? A1: They belong to the same general class (just like whales and bats are both mammals). —The right object is a modified version of the left object.—The left object is a modified version of the right object. —They are not related at all. Q2: How confident are you about your responses? A2: Very confident-confident-somewhat confident-somewhat unconfident-unconfident-very unconfident. Q3: How similar were the left and right objects? A3: Very similar-similar-somewhat similar-somewhat dissimilar-dissimilar-very dissimilar.

## Results

All raw data are publicly available at https://doi.org/10.5281/zenodo.7105616.

### Waterlily (serial homology)

*P1:* To test whether different participants perceive the same locations as corresponding (high response consistency), we calculated the average distance between the responses of different participants for each sample point (transparent ellipses in Fig. 2D–E). Then, we compared this to the average distance between randomly sampled locations anywhere within the contour of the test shape. Paired t-tests show that participants’ responses were much more consistent than random responses, in the condition without intermediates, 42.99 vs. 235.07 units, t(9) = − 18.56, *p < *0.001, as well as in the condition with intermediates, 99.80 vs. 235.07 units, t(9) = − 7.62, *p < *0.001. As a more conservative measure, we asked whether participants consistently identified different locations on the tepal when compared to neighboring but distinct dots on the stamen. For this purpose we determined whether the responses for one dot were closer to each other than to the median response for its neighboring sample point. This turned out to be the case in the condition without intermediates, 42.99 vs. 160.28 units, t(9) =  − 6.36, *p < *0.001, as well as in condition with intermediates, 99.80 vs. 169.33 units, t(9) =  − 2.26, *p* = 0.050.

Finally, participants were significantly more consistent in the condition without intermediates compared to the condition with intermediates, 42.99 vs. 99.80, t(9) =  − 4.67, *p* = 0.001.

*P2:* Next, we investigated whether participants’ correspondence judgments differed depending on whether or not they saw intermediate forms. In the condition *without* intermediates (Fig. 2E), participants were shown only the stamen (base stimulus with dot) and the tepal (test stimulus) (Fig. 2A). In the condition *with* intermediates (Fig. 2D), they were shown four stimuli simultaneously, i.e. base and test stimuli as well as two intermediate forms (Fig. 2B). For each sample point, we first calculated the distances between all individual responses per sample point in the condition without intermediates. We then compared this distance measure with two average (median) responses: (1) the average response in the condition without intermediates, as well as (2) the average response in the condition with intermediates. As a result, across sample points, the responses in the condition without intermediates were clearly much closer to their own average as to that of the condition with intermediates—showing how dissimilar responses were between the two conditions, 29.61 vs. 196.39 units, t(9) =  − 5.06, *p < *0.001. Generally, only a few dots are at similar locations in both conditions, namely the light yellow dots at the bottom of the tepal, while in the condition with intermediates the locations of all other points are shifted towards the tepal’s tip (Fig. 2D).

When comparing the speed between both conditions by calculating paired t-tests for the average response times per participant (across repetitions), we found that participants responded with similar speed, 5.68 vs. 6.10 s, t(9) =  − 0.42, *p* = 0.686. We also compared confidence and similarity judgements between conditions by paired t-tests, and found no significant difference for confidence, t(9) =  − 0.61, *p* = 0.555, but a significantly higher similarity in the condition with intermediates compared to the condition without intermediates, t(9) = 2.76, *p* = 0.022 (Fig. S2B,C). For identity judgements, we compared conditions with a two-sample t-test (because we allowed multiple responses per participant) and did not obtain a significant difference, t(24) = 0.71, *p* = 0.484 (Fig. S2D).

*P3:* Finally, we investigated whether the corresponding locations picked out by participants match the homologous locations as determined by experts and pre-evolutionary naturalists. This involved three steps. First, we determined the consistency of stamen-tepal homologies among experts in order to discern potential scientific uncertainties or disagreements. Expert identifications were significantly more consistent than random, 102.09 vs. 849.02 units, t(9) =  − 11.87, *p < *0.001 (Fig. S8A) and, across sample points, closer to each other than to the median response for neighboring sample points, 102.09 vs. 179.76 units, t(9) =  − 2.27, *p* = 0.050. Note, however, that in contrast to our expectations experts were not more consistent than naïve participants in the condition with intermediates, 102.09 vs. 99.80 units, t(9) = 0.13, *p* = 0.902. This relatively low level of expert consistency for neighboring sample points in the waterlily is illustrated and explained in Fig. S9: While some experts distributed the dots around the tepal (group 1, averaging experts 1 and 2; Fig. S9A), others grouped them into clusters (group 2, averaging experts 3, 4 and 5; Fig. S9B) or did not place all dots on the tepal (group 3, averaging experts 6 and 4; Fig. S9C). These differences reflect uncertainties about stamen-tepal homologies (see Discussion).

Second, we asked which expert group, if any, matched the views of pre-evolutionary botanists. Pre-evolutionary botanists believed that waterlily tepals are stamens with greatly expanded filaments (a stamen’s lower, stem-like half) and correspondingly contracted anthers (a stamen’s upper, pollen-bearing half). This hypothesis predicts that the blue and green dots at the lower end of the anther (Fig. 2C) correspond to locations near the tip of tepals, rather than to locations mid-way between a tepal’s tip and base (Supplementary Discussion, 1.1). We found that expert group 2 clustered the dots near the tip (Fig. S9B), showing that their homology judgments are in line with pre-evolutionary botanists.

Third, we compared the homology judgments of expert group 2 with the correspondence judgements of untrained participants. Participant responses in the condition *with* intermediates did not differ significantly from expert group 2, 74.73 vs. 110.10 units, t(9) =  − 1.92, *p* = 0.087, while diverging significantly from group 1, 74.73 vs. 154.75 units, t(9) =  − 4.42, *p* = 0.002 (Fig. 2D–F). This shows that participants allocated the dots similarly to those (and only those) experts whose homology judgments align with pre-evolutionary botanists. Furthermore, participant responses in the condition *without* intermediates differed significantly from group 2, 29.61 vs. 186.26 units, t(9) =  − 3.54, *p* = 0.006 as well as from group 1, 29.61 vs. 64.45 units, t(9) =  − 3.82, *p* = 0.004. This suggests that participants’ responses aligned with expert group 2 as the result of seeing the intermediate forms.

Furthermore, although participants’ responses were statistically different from expert group 1 in both without and with intermediate conditions, in the condition without intermediates the responses were still significantly closer to group 1 (cf. Figs. 2E and S9A) than to group 2 (64.45 vs. 186.26 units, t(9) =  − 3.11, *p* = 0.013). In contrast, participant’s results in the condition with intermediates were not closer to group 1 compared to group 2 (cf. Figs. 2D and S9B), 154.75 vs. 110.10 units, t(9) = 1.65, *p* = 0.133.

Note that we cannot make a sensible comparison between participants’ responses and expert group 3 (Fig. S9C) because we are missing expert correspondence judgements for most of the locations.

Finally, casual inspection of results of participants that were exclusively completing conditions without intermediates shows that differences between conditions were not resulting from mere repetition (Figs. S4 and S5).

*Conclusion.* In sum, (1) the responses of participants were highly consistent in both conditions, (2) the presence of intermediates affected participants’ average responses (dots cluster near the tip of the tepal), decreased their response consistency, and increased perceived similarity, and (3) the responses in the condition with intermediates matched the subset of experts (group 2) in line with the claims of pre-evolutionary botanists.

### Mammalian bones (specific homology)

*P1:* The consistency among participants’ responses was tested by calculating the average distance between the responses of different participants for each sample point (transparent ellipses in Fig. 3D,E). We then compared this measure to the average distance between randomly sampled locations within the contour of the test shape. Response consistency was much higher than random, in the condition without intermediates, 176.40 vs. 884.75 units, t(9) =  − 30.49, *p < *0.001, as well as in the condition with intermediates, 140.90 vs. 884.75 units, t(9) =  − 41.43, *p < *0.001. However, the more conservative measure of testing whether participants’ responses for a sample point were closer to each other than to the median response for its neighboring sample point failed to reach significance in the condition without intermediates, 176.40 vs. 262.79 units, t(9) =  − 1.66, *p* = 0.131, as well as in the condition with intermediates, 140.90 vs. 249.22 units, t(9) =  − 2.16, *p* = 0.059. This can also be seen in Fig. 3D,E: Although the dots are distributed along the arm bones, and some are therefore far apart, they occur in groups with overlapping ellipses.

Also, participants were significantly less consistent in the condition without intermediates compared to with intermediates, 176.40 vs. 140.90, t(9) = 5.35, *p < *0.001.

*P2:* We investigated whether participants’ correspondence judgments in the condition without intermediates were significantly different from their responses in the condition with intermediates. In the condition *without* intermediates (Fig. 3E), participants were shown only the bones of the dolphin’s pectoral fin (base stimulus with dot) and the monkey’s arm (test stimulus) (Fig. 3A). In the condition *with* intermediates (Fig. 3D), they were shown four stimuli simultaneously, i.e. base and test stimuli as well as forelimb bones of the fin whale and otter, respectively (Fig. 3B). For each sample point, we first calculated the distances of all individual responses in the condition without intermediates. This measure was then compared with two average (median) responses: (1) the average response in the condition without intermediates, as well as (2) the average response in the condition with intermediates*.*As a result, across sample points, the responses in the condition without intermediates are as close to their own average as to that of the condition with intermediates—showing how similar responses were in both conditions, 149.77 vs. 145.52 units, t(9) = 2.63, *p* = 0.028. For instance, the locations of the light blue and purple dots at the top of the monkey’s humerus in the condition without intermediates closely match their locations in the condition with intermediates (Fig. 3D,E).

When comparing the speed between both conditions, we found that participants responded markedly slower in the condition without intermediates, 7.33 vs. 5.83 s, t(9) = 2.68, *p* = 0.025. For confidence and similarity judgements, we compared conditions by paired t-tests, and found higher confidence for the condition with intermediates compared to without intermediates, t(9) = 3.87, *p* = 0.004, but no difference in similarity between both conditions, t(9) = 1.86, *p* = 0.096 (Fig. S3B,C). Also, for identity judgements, we compared conditions with a two-sample t-test (because we allowed multiple responses per participant), but did not find a significant difference, t(25) = 0.38, *p* = 0.710 (Fig. S3D).

*P3:* We determined whether the corresponding locations picked out by participants matched the homologous locations as determined by experts and pre-evolutionary naturalists. We first determined the consistency of dolphin-monkey homologies among our 4 experts (Fig. 3F). Expert identifications were highly consistent. That is, they were significantly more consistent (1) than random, 61.77 vs. 931.77 units, t(9) =  − 11.90, *p < *0.001 (Fig. 3F) and across sample points closer to each other than to the median response for neighboring sample points, 61.77 vs. 236.45 units, t(9) =  − 3.56, *p* = 0.002. Also, experts were more consistent (2) as compared to participants in the condition with intermediates, 61.77 vs. 140.904 units, t(9) =  − 2.42, *p* = 0.038, as well as in the condition without intermediates, 61.77 vs. 176.40 units, t(9) =  − 2.83, *p* = 0.020.

Next, we investigated the history of arm bone homologies in order to determine whether the homologies identified by our experts matched the views of pre-evolutionary anatomists. Many of the anatomical features of the base and test stimuli had been identified by the first decade of the nineteenth century (Supplementary Discussion, 1.2). The round upper end of the humerus, for example, was recognised as the same structure in dolphins, monkeys, and other mammals (as the ‘head’ of the humerus). Our experts homologized the same structures. For instance, they placed the purple and light-blue dots visible on the head of the dolphin’s humerus (Fig. 3C) on the head of the monkey’s humerus (Fig. 3F).

Finally, we compared the homology judgments of our experts with the correspondence judgements of untrained participants. For this we compared the distance between participant responses in the condition with intermediates (same information as experts) to (1) the average response in the condition with intermediates, and (2) the average expert response. Indeed, we find that the responses in the condition with intermediates are as close to their average as to the expert average—showing how similar responses of naïve participants and experts were, 118.49 vs. 110.27 units, t(9) = 0.66, *p* = 0.657 (cf. Fig. 3D,F).

Casual inspection of results of participants that were exclusively completing conditions without intermediates shows that differences between conditions were not resulting from mere repetition (Figs. S6, S7).

*Conclusion.* In sum, (1) the responses of participants were consistent in both conditions, but less so as for the waterlily (indicated by a more conservative test), (2) the presence of intermediates did not affect average responses (but significantly increased response speed, consistency, and perceived confidence), and (3) participants’ responses matched the homologous locations identified by experts.

## Discussion

### Participants used general human shape-matching abilities

Participants completed their responses quickly and consistently in both conditions and for both types of organs. They decided within seconds which location in the test stimulus corresponded to the marked location in the base stimulus. Furthermore, their responses were highly consistent, i.e. different participants identified similar locations as “corresponding” (P1), in line with previous findings in cognitive psychology (e.g.,^17,18^). Yet participants were not trained in plant or animal morphology. This suggests that participants’ responses resulted from the general human ability to find visual correspondences between differently shaped objects. Both the existence and sophistication of this ability are well established in cognitive psychology and linked to notions where shape is represented at many different levels simultaneously (e.g.,^25–27^) and is understood by parsing and interpreting its causally significant features^28–33^. For example, by representing shapes in terms of their medial axis or “skeleton”^34–37^, we might identify different parts but also establish correspondences between similar shapes (e.g.,^38,39^). Note that participants were less consistent when establishing correspondences for the mammalian bones compared to the waterlily, which might reflect higher demands of these particular stimuli (e.g., lower shape similarity in specific vs. serial homology, or more complex 3D structure of bones compared to floral organs).

Both morphometrics—the scientific, quantitative analysis of form—and the dot-matching paradigm employ dots to mark locations on biological structures. However, morphometrics uses them to measure biological objects (e.g.^40^), whereas dot-matching tasks measure correspondence judgments about these objects. Furthermore, geometric morphometrics requires marking anatomically corresponding positions in order to compare homologous structures^41^. The dot-matching paradigm does not make this requirement, and it is contentious among morphometricians whether it should be a requirement for all morphometric approaches (e.g.^42,43^). Future studies might explore the relation between morphometrics and the dot-matching paradigm.

### Shape-matching abilities underpin historical homology claims

The locations participants judged as “corresponding” matched the parts that pre-evolutionary naturalists believed to be identical (P3). First, contemporary observers agreed with the experts in this study, or at least with a subset of them. The judgments about flower organs matched expert group 2 but not others (see Waterlily (serial homology), P3). With respect to arm bones, the corresponding locations identified by participants were statistically indistinguishable from the homologous locations determined by four experts (see Mammalian bones (specific homology), P3). Second, the experts (or the relevant subset of experts) agreed with the views of pre-evolutionary naturalists (Supplementary Discussion, 1.1, 1.2). The forelimb parts in dolphins and monkeys, which early nineteenth century anatomists believed to be identical, are also the parts that experts in this study consider homologous (see Mammalian bones (specific homology), P3). Similarly, late eighteenth century botanists held that the tip of a waterlily’s petal is a transformed anther, and only these parts are homologous according to expert group 2 (see Waterlily (serial homology), P3). Thus, contemporary observers picked out the same corresponding parts as pre-evolutionary naturalists. Furthermore, since the observers in our study achieved this by spontaneous shape-matching (see Participants used general human shape-matching abilities), it is likely that pre-evolutionary naturalists did so, too.

An intuitive grasp of visual similarities has often been regarded as the “starting point” for comparative-anatomical research^14^ (p. 21) and, in particular, for conjecturing homologies (e.g.^13,15^). While this assumption was prima facie plausible, it remained untested and vague. It was not specified, for example, which psychological faculties may be involved, what it means to perceive correspondences (rather than objects), and what may be intuitive about this ability. Here, we provide experimental evidence for pre-evolutionary naturalists relying on a specific set of shared human visuo-cognitive skills, i.e. visual shape-matching. The speed and spontaneity of this ability renders the resulting correspondence judgments intuitive in the sense of arising without deliberate reflection. This, in turn, explains why pre-evolutionary morphologists could proceed for centuries “without method”^14^. Finally, reliance on shape-matching explains why pre-evolutionary naturalists could be successful in gaining valid insights. This is difficult to explain if their judgements were as “imprecise and subjective” as some suggest^15^. Psychological research has established that shape-matching is neither “imprecise” (low-resolution) nor “subjective” (inter-individually inconsistent). Our findings confirm this fact for two exemplary structures in the history of homology research.

We conclude that general human shape-matching abilities are sufficient to explain a major achievement of pre-evolutionary naturalists, i.e. recognising that two possibly very different looking organs are actually the same. Nothing more is required to identify even fine-grained correspondences, at least in two standard examples of homology. We do not conclude, however, that shape-matching is or was sufficient for recognising the correspondences as *homologies*, not even in Owen’s^23^ non-evolutionary sense of “special” and “serial” homology (the same organ in different species and in one individual, respectively, see Introduction). Owen distinguished homologous from analogous organs, i.e. organs with the same function, such as bird and butterfly wings. Yet in an earlier study by one of us^17^, participants matched bird and butterfly wings quickly and with high consistency (Fig. S1B and Supplementary Discussion, 1.3). Hence, shape-matching does not differentiate between homologous and analogous organs. Still, here we show that naïve participants matched the locations that pre-evolutionary naturalists considered identical, even before homologies and analogies began to be distinguished (in the 1820–1840s^8–10^). This fact is significant because participants might have chosen locations pre-evolutionary naturalists believed to be unrelated.

Our study concerns the task of distinguishing corresponding from non-corresponding *locations*, whereas pre-evolutionary naturalists had to distinguish, in addition, corresponding from non-corresponding *organs*. We restricted our experiments to the first task, ensuring that participants were unfamiliar with the compared structures, but suggest that it may have been a means of achieving the second task.

Unlike pre-evolutionary naturalists, our participants only saw images of objects, not the objects themselves. It is possible that real objects would introduce more sources of variability and potentially inconsistency between naïve participants. However, they would also provide more opportunities for observation. Together with our participants having less biological knowledge than pre-evolutionary naturalists, we think that these differences strengthen our conclusions. For they show that the shape-matching ability operates successfully with fewer observational opportunities and less biological training. Future studies might test whether other modes of presentation would affect the results (e.g., three-dimensional objects presented in virtual reality, or different orientations of object images).

Some homology judgments were found to be inconsistent, both among experts and between experts, current participants, and/or pre-evolutionary naturalists. Such inconsistencies have multiple reasons. Unlike participants who relied on the visible features of the images, and unlike pre-evolutionary naturalists who relied on the visible features of the actual bones and flower organs, we asked the experts to convey the current state of knowledge. Current homology criteria typically include features invisible to the naked eye, such as gene expression patterns in floral organs^44^. Furthermore, the discovery that floral organs are not homologous across all flowering plants casts doubts on stamen-tepal homologies^5,7,45^, especially in basal clades like the Nymphaeacea^7,46^. Different views among botanical experts are therefore to be expected. Expert inconsistencies about the homologies of forearm bones (circles in Fig. 3F) were probably caused by the pronation of the monkey’s forearm (due to its hand being placed on the ground): the radius crosses over the ulna and obscures parts easily visible in the dolphin.

### Seeing intermediate forms

When faced with differently shaped structures, pre-evolutionary naturalists commonly searched for intermediate forms^8,47^. For instance, Lawrence^48^ believed that species such as sea otters bridge the dissimilarities between the forelimb bones of whales and terrestrial mammals. Similarly, intermediate floral organs in *Nymphaea* were regarded as evidence for the fundamental identity between petals and stamens (Supplementary Discussion, 1.1).

In our experiments, seeing intermediates significantly affected responses in both floral organs and forelimb skeletons, albeit in different ways (Supplementary Discussion, 1.3). In forelimb bones, it did not change average responses, but instead increased their consistency and speed, together with perceived confidence (see Mammalian bones (specific homology), P2). These findings suggest that seeing intermediate bones solidified participants’ previous judgements, i.e. the correspondences that participants had identified in the condition without intermediates.

By contrast, seeing intermediate floral organs strongly affected average responses in the Waterlily (see Waterlily (serial homology), P2). In the condition without intermediates, participants responded that the locations in the stamen correspond to similarly spaced locations all around the tepal (Fig. 2E). But when seeing the intermediate forms, they responded that the dots on the stamen’s anther correspond to locations near the tip of the tepal (Fig. 2D). Seeing the intermediates also decreased consistency among participants. Apparently, seeing intermediate floral organs destabilized and altered, rather than confirmed, participants' previous correspondence judgments.

Overall, the presence of intermediates changed the correspondence judgments that untrained observers made in their absence. This finding suggests that seeing intermediates may have had the same effect on pre-evolutionary naturalists. That is, intermediates may have altered *pre-existing* correspondence claims, in addition to suggesting new homologies in the first place (as hypothesized by^14^).

## Conclusion

Our study suggests that pre-evolutionary naturalists might have discovered correspondences that underpin many homologies by general human shape-matching abilities. It also opens an experimental route to investigating these visuo-cognitive mechanisms, which sustained biological research for centuries.

## Supplementary Information

Supplementary Information.

## Data Availability

All raw data are publicly available at https://doi.org/10.5281/zenodo.7105616.
