# Supplementary material for: Homology judgements of pre-evolutionary naturalists explained by general human shape matching abilities
Source: Sci Rep. 2023 Jul 28;13:12269. doi: 10.1038/s41598-023-39036-2 (PMC10382571; doi:10.1038/s41598-023-39036-2)
Supplement: Supplementary file 1 — Supplementary Information. [file 41598_2023_39036_MOESM1_ESM.docx]

**Supplementary Information**

Homology judgements of pre-evolutionary naturalists explained by general human shape matching abilities

Ulrich E. Stegmann and Filipp Schmidt

Corresponding author: Filipp Schmidt

Email: [filipp.schmidt@psychol.uni-giessen.de](mailto:filipp.schmidt@psychol.uni-giessen.de)

**1. Supplementary discussion**

**1.1 The homology judgments of expert group 2 agree with pre-evolutionary botanists**

Our historical investigations show that the petaloid staminodes of *Nymphaea* spp. were discovered in the late 18^th^ century [1, 2]. Sowerby ([2] tab. 160) described the petals of *N. alba* as “gradually lessening and running into the stamina, so that the line of distinction can hardly be drawn between them”. The existence of intermediate organs was soon interpreted as evidence for the fundamental identity of petals and stamens, both in *Nymphaea* spp. (e.g. [1, 3, 4]) and other taxa (e.g. [5] §47; [6]). Gray [3] described the identity of floral organs in terms of “homology”, albeit still in its pre-evolutionary sense. The foliar nature of petals and stamens was widely accepted by the mid-19^th^ century, with botanists mostly disagreeing over whether petals derive from stamens or the other way around [7].

In *N. alba*, the tepal (or ‘petal’ in the literature around 1800) is homogeneously white, whereas the stamen is separated into the yellow anthers and the thread-like filament. This raises the question as to which area of the tepal pre-evolutionary botanists regarded as corresponding to which part of the stamen. To the extent they described a series of intermediates, they agreed that the filament becomes larger towards the outer whorls, while the anther contracts until it entirely disappears. Correspondingly, they identified the petal with the *filament* of the stamen (for *Nymphaea* spp. see [1, 3, 4]; and eventually [7]; for other taxa see [5], and [6]). Voigt ([4] p. 81) stated it explicitly: “the Filamentum... proves itself quite clearly as a contracted petal”. Importantly, the filament-hypothesis (of pre-evolutionary botanists) has clear implications for the blue and green dots that mark the lower end of the anther in fully-formed stamens (Fig. 2C). Since the anthers contract at their lower ends, they drag that end upwards towards the tip of the intermediate organs. Consequently, the blue and green dots will move upwards, as well. Likewise, the other dots on the anther (of the fully-formed stamen) will cluster near the tip of the intermediates and, eventually, the tepal. This can be observed in expert group 2 (Fig. S9B).

Some of the botanists who identified the petal with the filament summarized their findings by relating petals to entire stamens, rather than to filaments only (“stamens transition into petals”, [1] p.111; “stamens... and petals are the same organs”, [4] p. 14). We can employ this fact to construct an alternative hypothesis about petal-stamen transitions: filament and anther expand in equal proportion towards the outer whorls while also transforming their distinctive colors and textures into that of the petal. The whole stamen-hypothesis predicts that the anther’s lower end does not move upwards, but rather remains in place, albeit in a transformed state. Therefore, the blue and green dots would remain where they were in the stamen, i.e. half-way between the top and bottom of the organ. This can be observed in expert group 1 (Fig. S9A). We articulate the whole-stamen-hypothesis for contrastive purposes only. It was rarely defended historically (but see [8], §83).

**1.2 Pre-evolutionary anatomists discovered** **contemporary arm bone homologies**

In 1680, Tyler recognised the similarities between the bones of a human arm and the pectoral fin of a porpoise [9]. He named the whale bones according to what he believed were the same bones in humans. For instance, he matched the upper, single arm bone in humans (then already called ‘humerus’) to the upper, single bone in the porpoise’s fin and called it ‘humerus’. Similarly for the two lower arm bones (‘ulna’ and ‘radius’). Furthermore, Tyler correctly depicted the positions of these bones relative to shoulder blades and hands. By the early 19th century, comparative anatomists had discerned many additional anatomical details, which are accepted today. For example, Cuvier [10, Lecture IV, Articles III and V] described parts of the upper (e.g. head, anatomical neck, greater and lesser tubercle) and lower extremity of the human humerus (e.g. medial and lateral epicondyles) as well as parts of the human ulna that articulate with the humerus (e.g. semilunar notch, olecranon, and coronoid process). Cuvier distinguished and named the same features in many other vertebrates, including monkeys and whales, while noting differences in size and shape. Thus, pre-evolutionary anatomists had realized that, say, the round upper end of the monkey humerus is the same structure as the round upper end of whale humerus (i.e. its ‘head’).

**1.3 Differences in the effects of intermediates**

The differences in the effect of intermediates between forelimb bones and floral organs can be explained by a two-step model of visual processing. Previous studies by one of us [11, 12] suggest that the visual-cognitive system (1) first identifies corresponding salient landmarks between two objects and then (2) employs these landmarks to establish corresponding locations elsewhere. The first step, landmark selection, will depend on the familiarity and similarity of the two objects. When they are unfamiliar but similar, observers can find geometrically salient features (e.g., by rotating a simple shape as in Fig. S1A). However, when objects are not similar enough to find corresponding geometrical features, observers can base their correspondence judgements on semantic information instead to find familiar elements that correspond (e.g., recognize the two shapes on the butterfly and owl as wings,the wings of a butterfly and an owl as in Fig. S1B). Accordingly, humans infer corresponding locations on unfamiliar shapes from corresponding geometric shape features; for familiar shapes with (sufficiently) similar part organization, they base their correspondence judgements on semantic information instead, such as recognizing the two shapes on the butterfly and owl as wings.


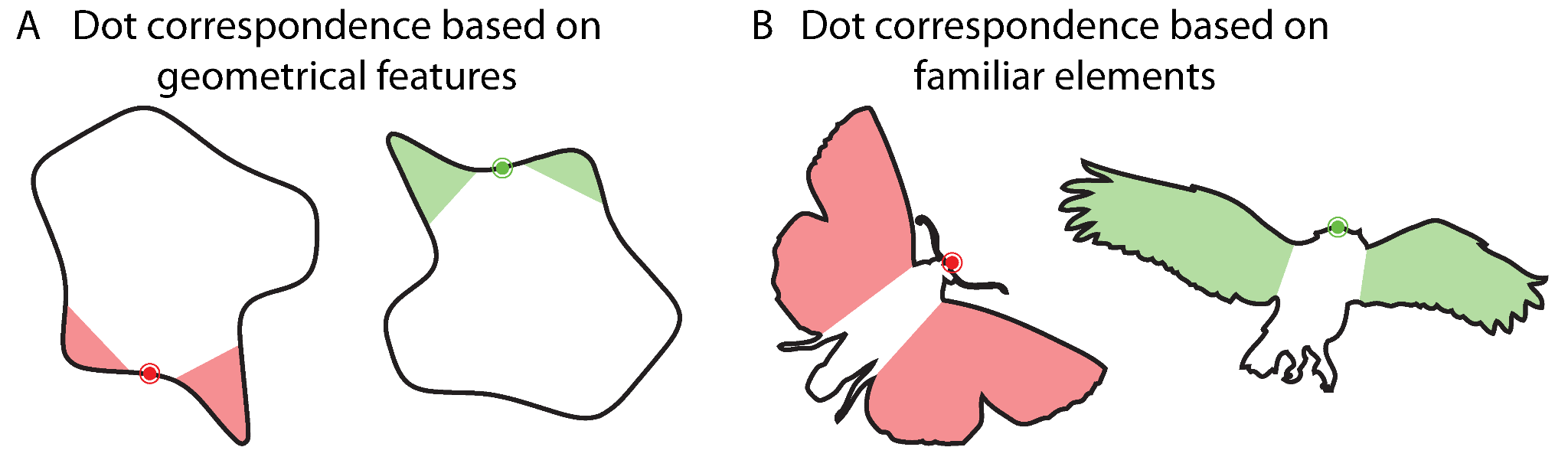


**Fig. S1.** Examples of how humans establish visual correspondences between locations (red to green dots) on differently shaped objects, based on landmarks such as (A) salient geometrical features [12] or (B) familiar elements [11]. Butterfly silhouette by shutterstock.com/ntnt, owl silhouette by yayimages.com/Perysty.

Since our participants were unfamiliar with the particular bones and floral organs, they probably relied on geometrically salient features to identify the landmarks. We hypothesize that presenting intermediate shapes in forelimbs did not affect landmark selection (part decomposition) and therefore did not change the correspondence judgments overall. In both the without and with intermediate conditions, part decomposition seems to include ignoring the “finger” and “shoulderblade” bones (probably by estimating the overall “structure” of the two bones).

In flower organs, we hypothesize that without intermediates participants used features such as width and curvature first to decompose the base stimulus (stamen) into two equally sized upper and lower parts and then projected this division onto the test stimulus, resulting in a similar distribution of dots around the contour. However, seeing the intermediates caused participants to decompose the base test stimulus into a smaller upper and a larger lower part. Consequently, they select different parts of the test stimulus as corresponding: All dots falling unto the upper part of the base stimulus are now projected unto the top 20% of the test stimulus, resulting in these dots moving higher up.

**2. Supplementary figures**


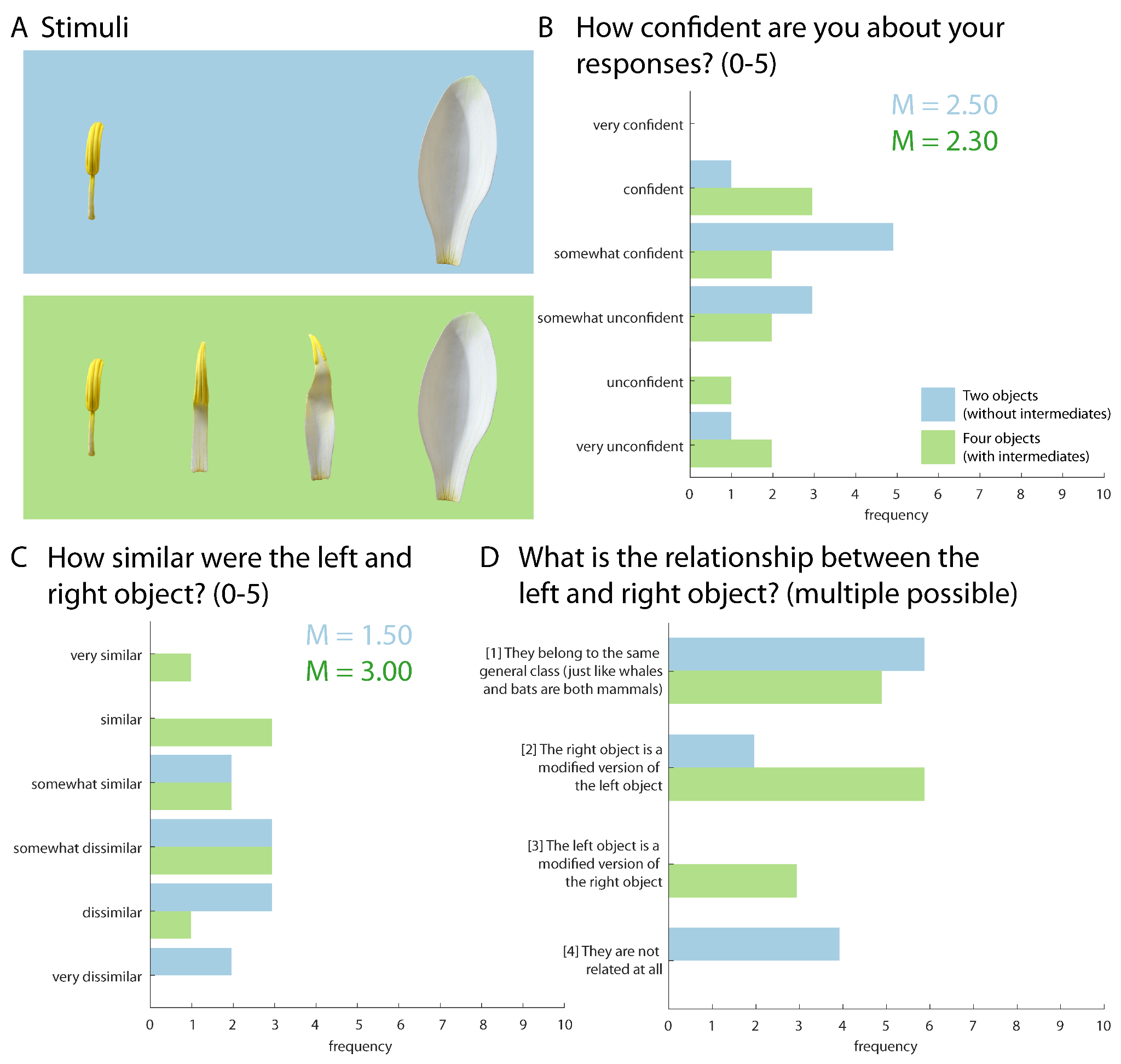


**Fig. S2.** Stimuli and multiple-choice results for the White Waterlily (*Nymphaea alba* L.). (A) Stimuli in the two conditions of our experiment (see Fig. 2A, B). (B) Response frequencies for confidence judgements without (blue) and with intermediates (green). (C) Response frequencies for similarity judgements without (blue) and with intermediates (green). (D) Response frequencies for identity judgements without (blue) and with intermediates (green). For image sources and rights see Fig. 2.


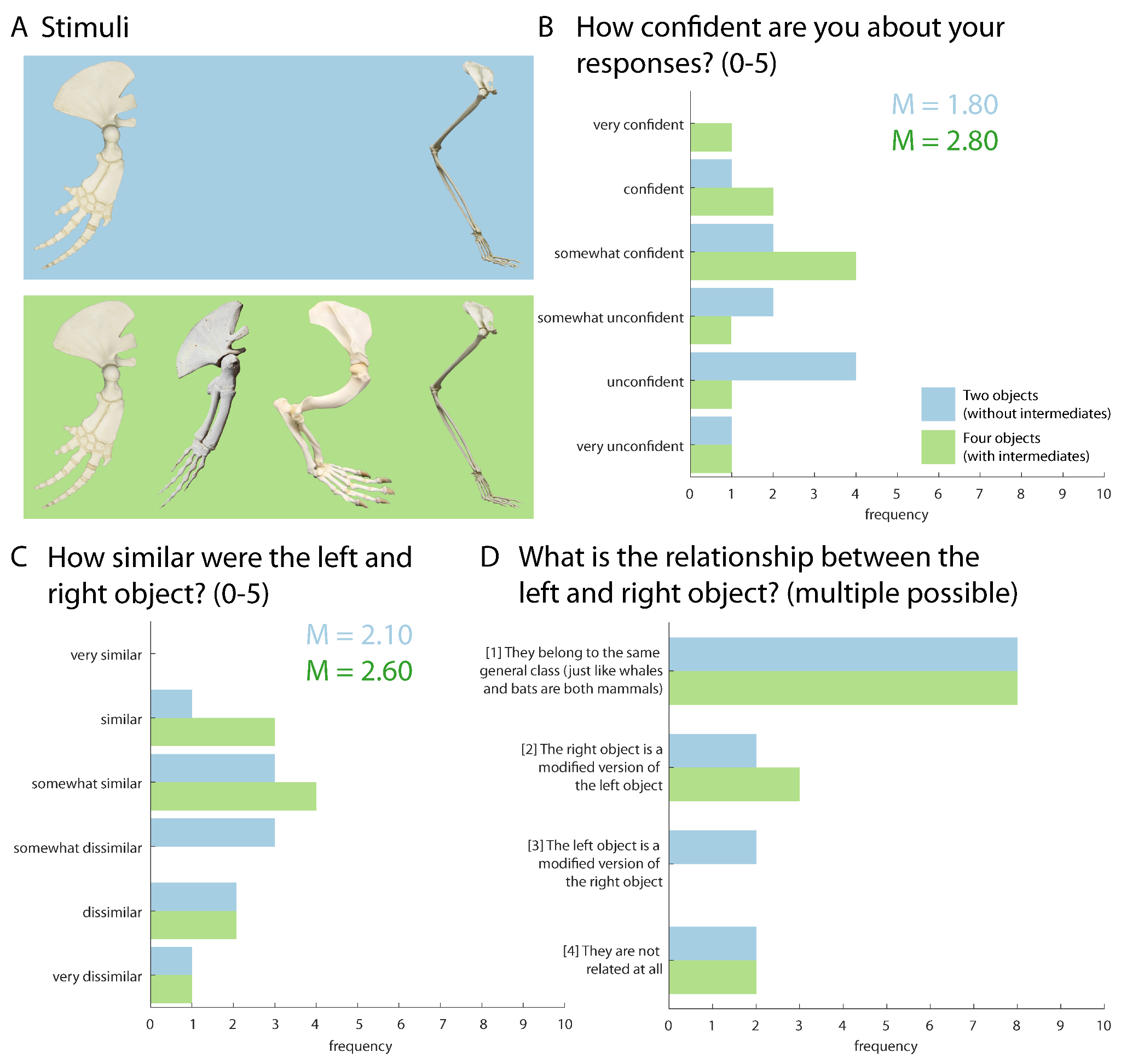


**Fig. S3.** Stimuli and multiple-choice results for mammalian bones stimuli. (A) Stimuli in the two conditions of our experiment (see Fig. 3A, B). (B) Response frequencies for confidence judgements without (blue) and with intermediates (green). (C) Response frequencies for similarity judgements without (blue) and with intermediates (green). (D) Response frequencies for identity judgements without (blue) and with intermediates (green). For image sources and rights see Fig. 3.


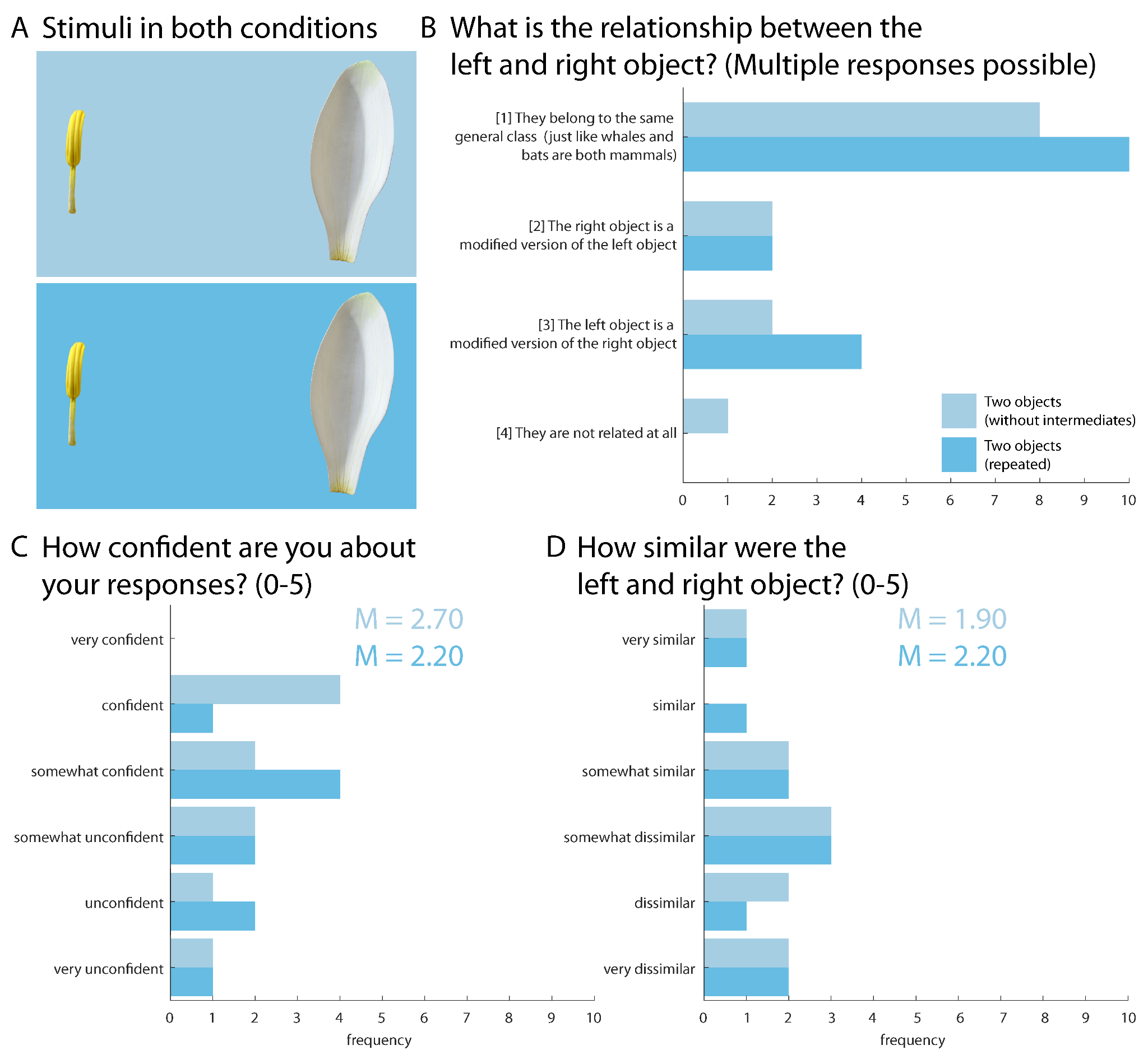


**Fig. S4.** Multiple-choice results for waterlily stimuli without intermediates (light blue) and repeated presentation without intermediates (dark blue). For other details and image sources see Fig. 2.


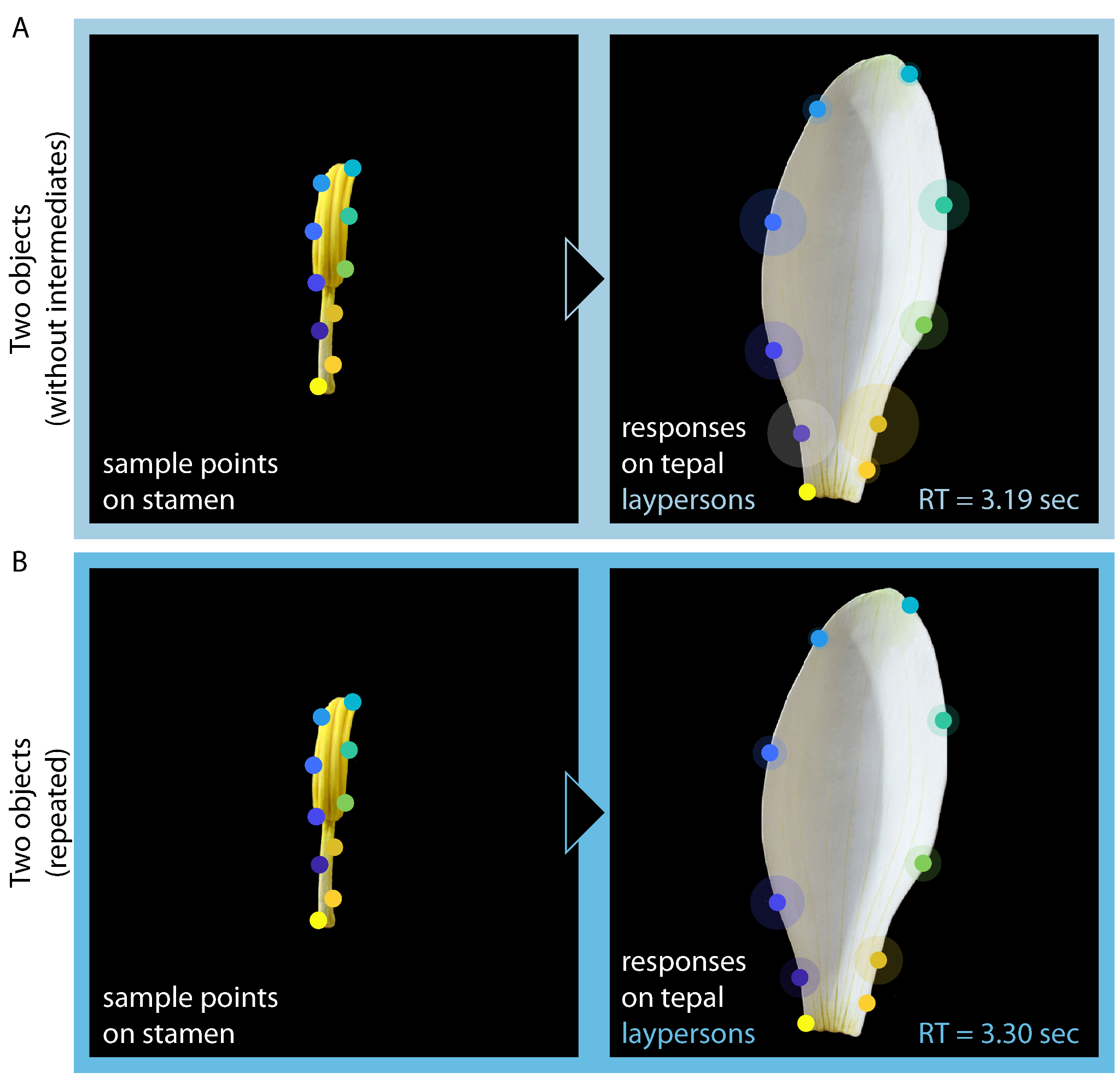


**Fig. S5.** Dot matching results for waterlily stimuli (A) without intermediates and (B) repeated presentation without intermediates. For details and image sources Fig. 2.


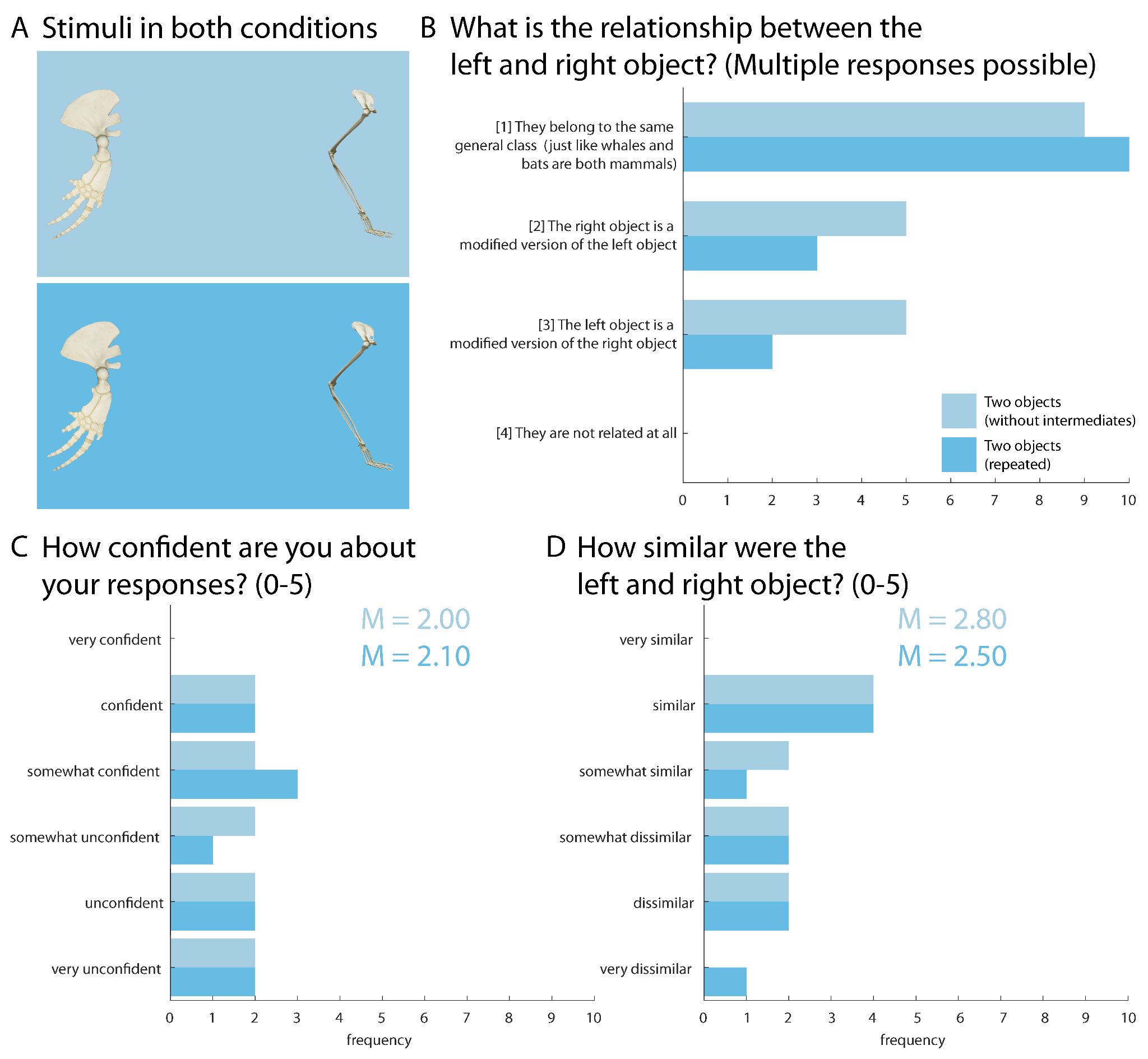


**Fig. S6.** Multiple-choice results for mammalian bones stimuli without intermediates (light blue) and repeated presentation without intermediates (dark blue). For other details and image sources see Fig. 3.


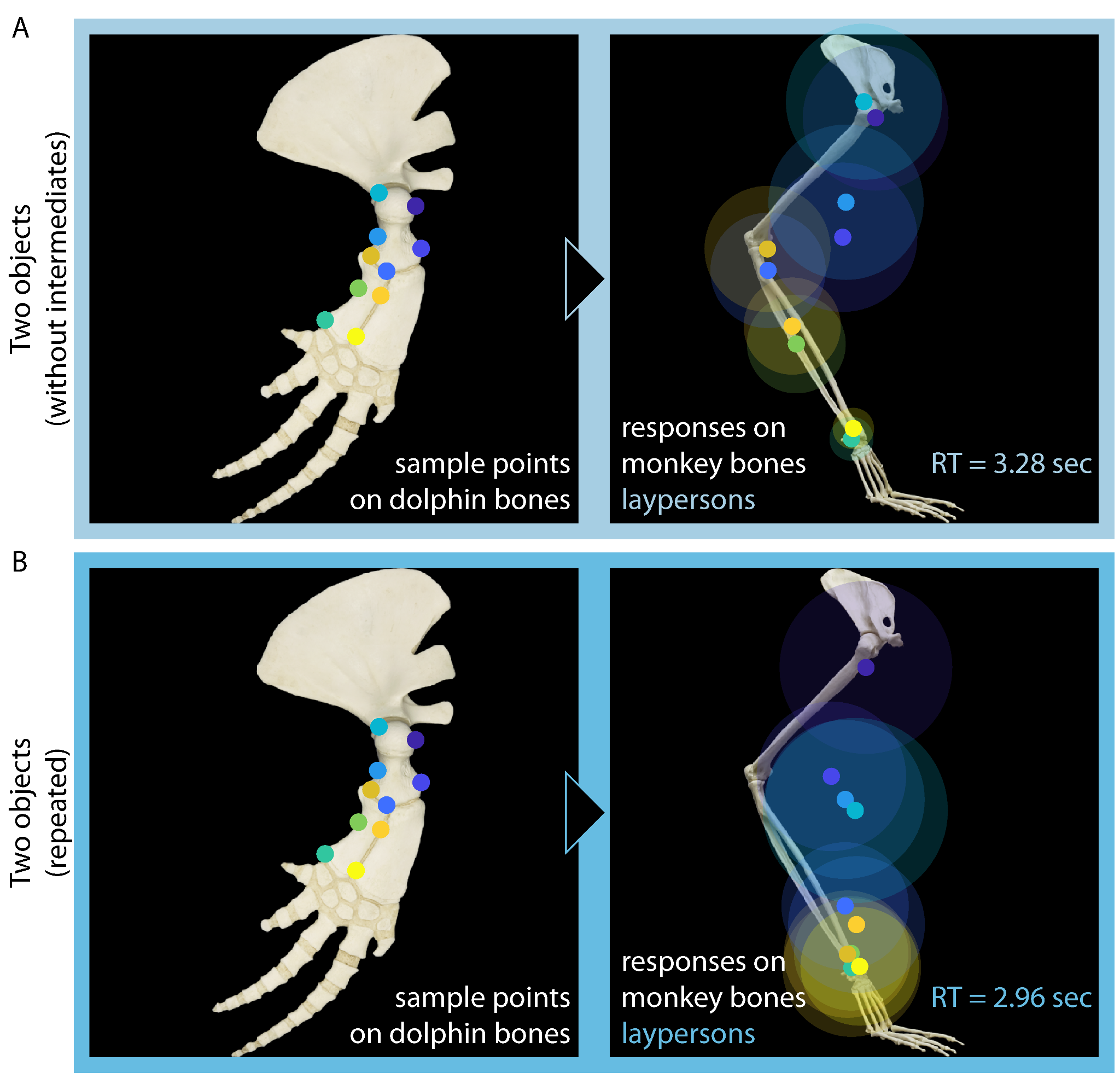


**Fig. S7.** Dot matching results for mammalian bones stimuli (A) without intermediates and (B) repeated presentation without intermediates (control). For details and image sources Fig. 3.


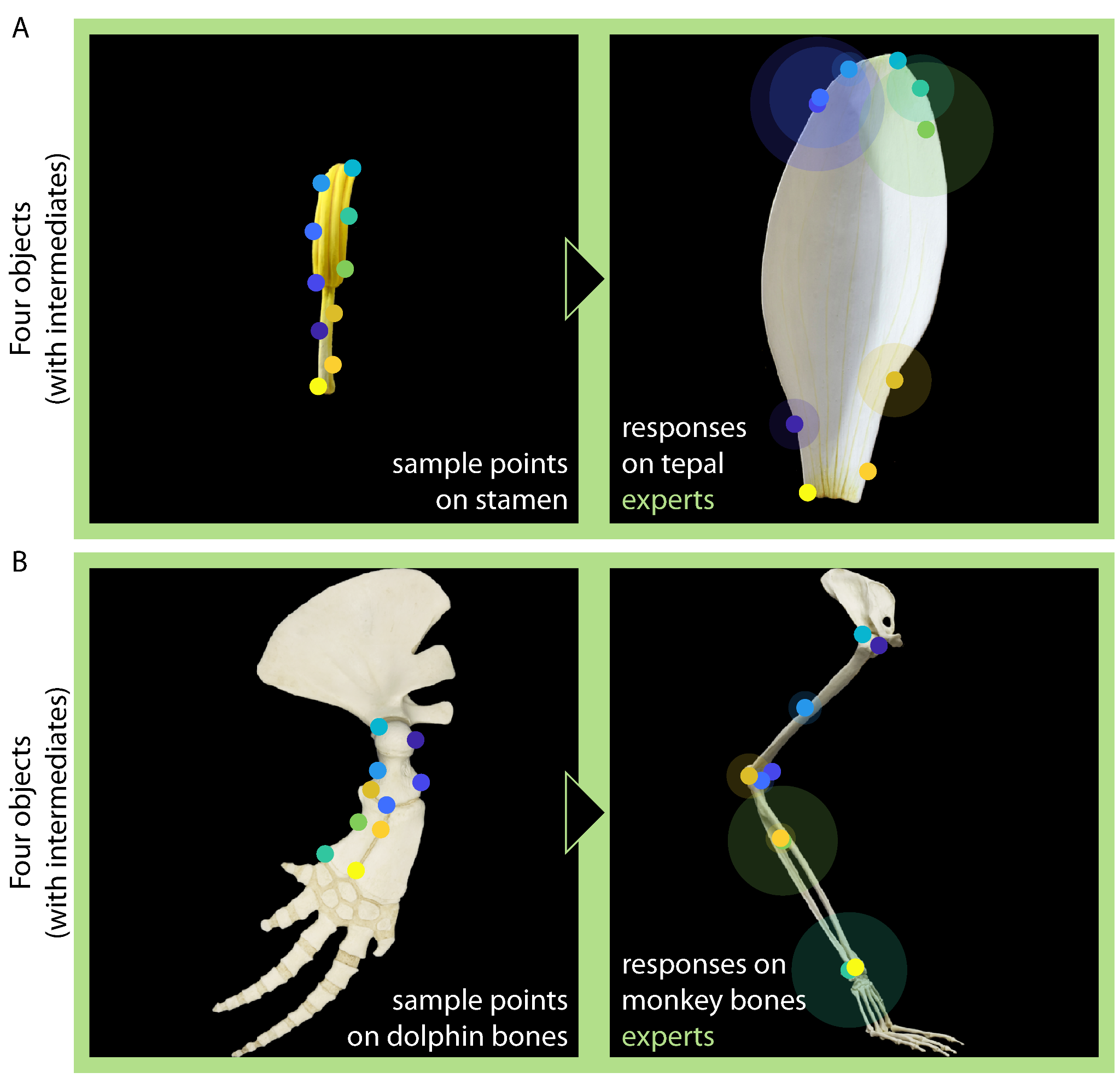


**Fig. S8.** Dot matching results of experts for (A) waterlily and (B) mammalian bones stimuli. Individual expert responses for waterlily are shown in Fig. S9. For details and image sources see Figs. 2 and 3.


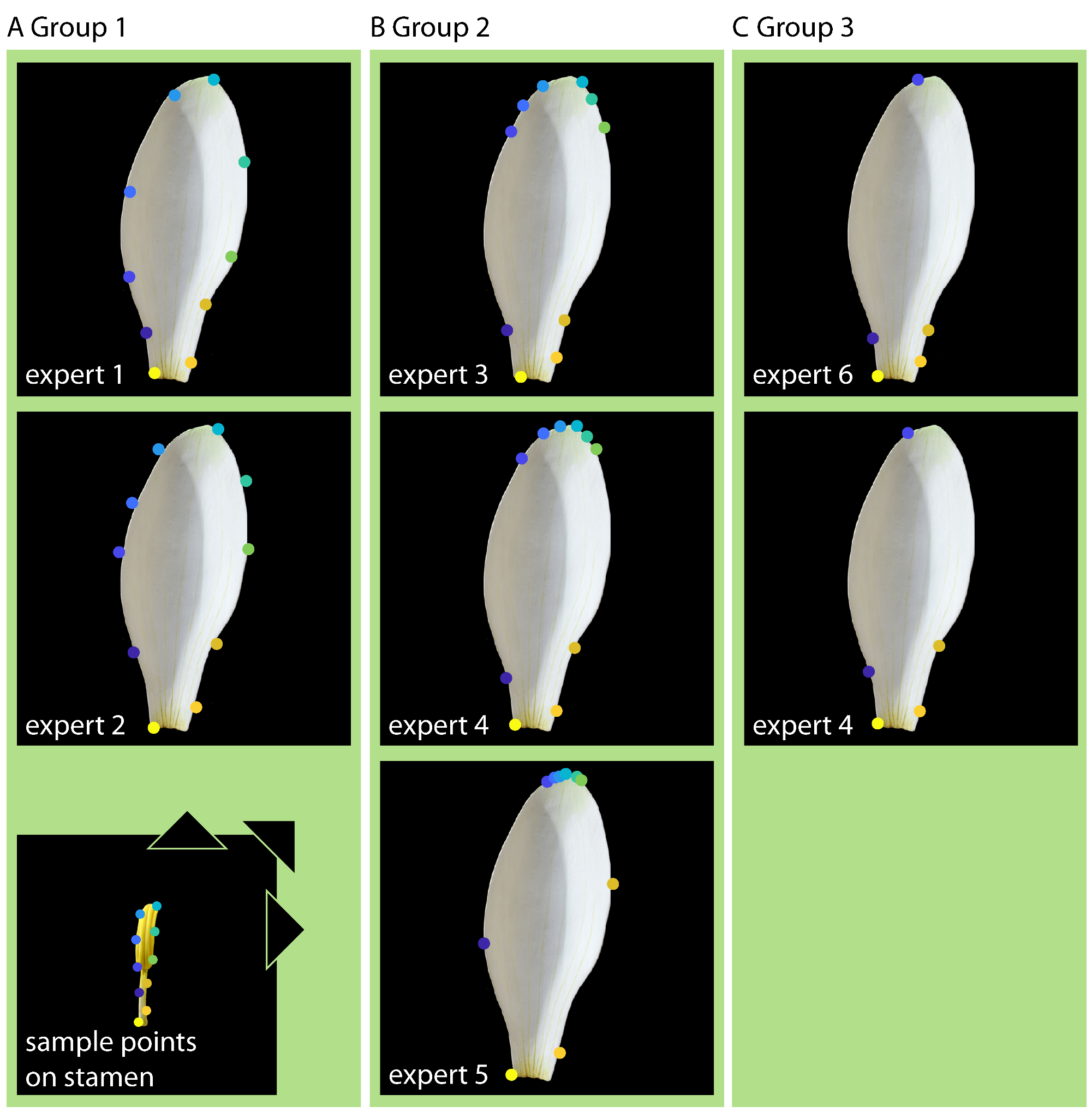


**Fig. S9.** Dot matching results of 6 experts for waterlily. We sorted experts by their response behavior into three groups based on correspondence according to (A) position (original; group 1), (B) position (intermediate, group 2), and (C) organ identity (group 3). Group 2 is in accordance with the responses of pre-evolutionary botanists. Note that one expert (“Expert 4”) provided responses based on position (intermediate) as well as on organ identity (B, C). For image sources see Fig. 2.

**Supplementary References**

1. Batsch, A. J. G. K. *Botanik für Frauenzimmer und Pflanzenliebhaber, welche keine Gelehrten sind* (Industrie-Comptoir, Weimar, 1795).
2. Sowerby, J. *English botany or Coloured figures of British plants* 3rd edn, Vol. 3 (R. Hardwicke, 1863).
3. Gray, A. *The Botanical Text-Book, or An Introduction to Scientific Botany, Both Structural and Systematic* 4th edn (G. P. Putnam, New York, 1853).
4. Voigt, F. S. *System der Botanik* (Akademische Buchhandlung, Jena, 1808).
5. Goethe, J. W. v. *Versuch die Metamorphose der Pflanzen zu erklären* (C. W. Ettinger, Gotha, 1790).
6. De Candolle, A. P. *Théorie élémentaire de la botanique* (Déterville, Paris, 1813).
7. Troll, W. *Zur Frage nach der Herkunft der Blumenblätter*. Flora oder Allgemeine Botanische Zeitung 122:57–75. <https://doi.org/10.1016/s0367-1615(17)31002-9> (1927).
8. Kieser, D. G. *Aphorismen aus der Physiologie der Pflanze* (H. Dieterich, Goettingen,1808).
9. Tyson, E. *Phocæna, or, The anatomy of a porpess, dissected at Gresham Colledge* (B. Tooke, London, 1680).
10. Cuvier, G. *Leçons d’anatomie comparée* (Crochard, Paris, 1805).
11. Schmidt, F., Kleis, J., Morgenstern, Y. et al. *The role of semantics in the perceptual organization of shape*. Sci Rep 10:22141. <https://doi.org/10.1038/s41598-020-79072-w> (2020).
12. Schmidt, F. & Fleming, R.W. *Visual perception of complex shape-transforming processes*. Cogn Psychol 90:48–70. <https://doi.org/10.1016/j.cogpsych.2016.08.002> (2016)
